# In Vitro and In Silico Evaluation of the Antimicrobial and Antioxidant Potential of *Thymus pulegioides* Essential Oil

**DOI:** 10.3390/antiox11122472

**Published:** 2022-12-15

**Authors:** Călin Jianu, Laura-Cristina Rusu, Iulia Muntean, Ileana Cocan, Alexandra Teodora Lukinich-Gruia, Ionuț Goleț, Delia Horhat, Marius Mioc, Alexandra Mioc, Codruța Șoica, Gabriel Bujancă, Adrian Cosmin Ilie, Delia Muntean

**Affiliations:** 1 Faculty of Food Engineering, University of Life Sciences “King Michael I” from Timisoara, Calea Aradului 119, 300645 Timisoara, Romania; 2 Faculty of Dental Medicine, “Victor Babes” University of Medicine and Pharmacy, 2nd Eftimie Murgu Square, 300041 Timisoara, Romania; 3 Multidisciplinary Center for Research, Evaluation, Diagnosis and Therapies in Oral Medicine, “Victor Babes” University of Medicine and Pharmacy, Spl. Tudor Vladimir escu 14A, 300173 Timisoara, Romania; 4 OncoGen Centre, County Hospital “Pius Branzeu”, Blvd. Liviu Rebreanu 156, 300736 Timisoara, Romania; 5 Faculty of Economics and Business Administration, West University of Timisoara, 300233 Timisoara, Romania; 6 Faculty of Medicine, “Victor Babes” University of Medicine and Pharmacy, 2nd Eftimie Murgu Square, 300041 Timisoara, Romania; 7 Faculty of Pharmacy, “Victor Babeș” University of Medicine and Pharmacy, Eftimie Murgu Square 2, 300041 Timisoara, Romania; 8 Center for Translational Research and Systems Medicine, “Victor Babes” University of Medicine and Pharmacy, 2nd Eftimie Murgu Square, 300041 Timisoara, Romania; 9 Multidisciplinary Research Center on Antimicrobial Resistance, “Victor Babes” University of Medicine and Pharmacy, 2nd Eftimie Murgu Square, 300041 Timisoara, Romania

**Keywords:** *Thymus pulegioides*, essential oil, antimicrobial activity, antioxidant activity, molecular docking

## Abstract

The study was designed to analyze and evaluate the antioxidant and antibacterial properties of the essential oils of *Thymus pulegioides* L. grown in Western Romania. *Thymus pulegioides* L. essential oil (TPEO) was extracted by steam distillation (0.71% *v/w*) using a Craveiro-type apparatus. GC-MS investigation of the TPEO identified 39 different compounds, representing 98.46% of total oil. Findings revealed that thymol (22.89%) is the main compound of TPEO, followed by *para*-cymene (14.57%), thymol methyl ether (11.19%), isothymol methyl ether (10.45%), and beta-bisabolene (9.53%). The oil exhibits good antibacterial effects; *C. parapsilosis, C. albicans*, *S. pyogenes*, and *S. aureus* were the most sensitive strains. The antioxidant activity of TPEO was evaluated by peroxide and thiobarbituric acid value, 1,1-diphenyl-2-picrylhydrazyl radical (DPPH), [2,2′-azinobis(3-ethylbenzothiazoline-6-sulfonic acid) diammonium] (ABTS) radical scavenging assay, and beta-carotene/linoleic acid bleaching testing. The antioxidative data recorded reveal, for the first time, that TPEO inhibits primary and secondary oxidation products, in some particular conditions, better than butylated hydroxyanisole (BHA) with significant statistical difference (*p* < 0.05). Moreover, TPEO antioxidant capabilities in DPPH and ABTS assays outperformed alpha-tocopherol (*p* < 0.001) and delta-tocopherol (*p* < 0.001). Molecular docking analysis revealed that one potential target correlated with the TPEO antimicrobial activity was d-alanine-d-alanine ligase (DDl). The best scoring ligand, linalyl anthranilate, shared highly similar binding patterns with the DDl native inhibitor. Furthermore, molecular docking analysis also showed that the main constituents of TPEO are good candidates for xanthine oxidase and lipoxygenase inhibition, making the essential oil a valuable source for protein-targeted antioxidant compounds. Consequently, TPEO may represent a new potential source of antioxidant and antibacterial agents with applicability in the food and pharmaceutic industries.

## 1. Introduction

Food additives are substances deliberately added to food products for technological purposes (for example, preservation) that can be incorporated directly or indirectly into them, affecting their characteristics [1]. Additives such as butylated hydroxyanisole (BHA), butylated hydroxytoluene (BHT), gallic acid esters (e.g., propyl gallate, octyl gallate, and dodecyl gallate), sorbates, sulfites, and benzoates are widely employed to prolong the shelf-life of foodstuffs and to improve their overall quality and safety [2,3]. However, despite their benefits, food consumers need to be aware that some synthetic additives can be responsible for several life-threatening conditions (e.g., allergic reactions, asthma, nausea, or in some circumstances, even carcinogenesis) [4,5,6]. Therefore, the food industry has now focused on discovering natural alternatives to synthetic additives to manage food safety preservation without threatening consumers’ health.

The plant kingdom represents a rich source of valuable natural bioactive compounds with multiple applications in the food and pharmaceutical industries. For instance, natural extracts such as essential oils (EOs) could be an appropriate alternative to synthetic antimicrobial medicines, especially antibiotics [7,8,9]. Furthermore, the ability of EOs to inhibit the growth of undesirable microorganisms and lipid oxidation in foodstuffs could solve the substitution problem for synthetic additives and meet the increasing demand from food customers for developing “clean label” products [10].

The genus *Thymus* (Lamiaceae) comprises 215 species distributed all over the Eurasian continent, North and East Africa, and southern Greenland [11]. Among genus species, *Thymus vulgaris* L., *T. zygis* L., *T. serpyllum* L., and *T. pulegioides* L. are the most important commercial thyme species globally [12]. *T. pulegioides* is found throughout Europe [11], and due to the aromatic and antimicrobial properties of its oil [13,14,15,16], it is used as a medicinal plant, cosmetic and spice [16,17,18]. In Romania, the *Thymus* genus includes one cultivated aromatic species (*Thymus vulgaris*) and another 18 wild species (e.g., *T. pannonicus*, *T. glabrescens*, *T. pulegioides*, *T. austriacus*) [19,20]. 

Chemotypes are particularly common in Lamiaceae, also in thyme, being genetically determined and characterized by differing compositions of EOs [21,22]. For instance, *T. pulegioides* have previously reported six chemotypes: linalool (L), geranial/geraniol/neral (G/G/N), thymol (T), carvacrol/γ-terpinene/*p*-cymene (C/γT/*p*C), thymol/carvacrol/γ-terpinene/*p*-cymene (T/C/γT/*p*C), and α-terpenyl acetate [21,23,24]. This chemical diversity can influence the *T. pulegioides* essential oil (TPEO) biological activity and generate various applications: the monoterpenic phenols (e.g., carvacrol and thymol) have strong and wide-spectrum antimicrobial and antifungal properties with multiple applications in the food and pharmaceutical industry [25,26,27]; the acyclic monoterpene alcohols (e.g., geraniol and linalool) are fragrance ingredients with application in cosmetic and food industries, as insect repellents, and as pharmaceutical ingredients [28,29,30]; while α-terpinyl acetate exhibits antimicrobial properties [31]. However, to our knowledge, no studies regarding TPEO’s antioxidant activity have been mentioned in the scientific literature.

Consequently, the main aim of this research was to analyze: (i) the chemical profile of TPEO by GC-MS technique; (ii) the antimicrobial and antioxidant properties of the TPEO; and (iii) a potential protein-targeted mechanism of action correlated with the biological antimicrobial and antioxidant activity of TPEO, to find new sources of green preservative and/or antioxidant with applicability in the food and pharmaceutic industries.

## 2. Materials and Methods

### 2.1. Raw Material and Chemicals

Samples of *T. pulegioides*’ aerial parts (stems, leaves, and flowers) were harvested manually from the Ludești de Jos village, Hunedoara County (Romania) in August of 2020 (45°43′5″N 23°10′21″E), authenticated and deposited in the herbarium of the Faculty of Agronomy, University of Life Sciences “King Michael I” from Timișoara under accession no. VSNH.BUASTM-101/2. A total of 3.0 L cold-pressed sunflower oil was bought from the local market (1.8 meq · kg^−1^ initial peroxide value). In addition, Na_2_SO_4_ anhydrous, C_8_-C_20_ alkane standard mixture, chloroform, ethanol, hexane, methanol, thiobarbituric acid (TBA), butylated hydroxytoluene (BHT), butylated hydroxyanisole (BHA), 1,1-diphenyl-2-picrylhydrazyl radical (DPPH), 2,2′-azinobis (3-ethylbenzothiazoline-6-sulfonic acid) diammonium salt (ABTS), potassium persulfate (K_2_S_2_O_8_), and β-carotene were obtained from Sigma-Aldrich (Darmstadt, Germany).

### 2.2. Extraction of TPEO

The collected raw material was air-dried in the dark at room temperature (middle of 2020/beginning of 2021), then cut into approximately 2 cm long pieces, and stored in a paper bag until use. The dried plant material (100 g) was submitted to steam distillation for 4 h at 100 °C temperature using a Craveiro-type apparatus [32]. A glass boiler (3000 mL) filled with water (refilled as needed) and heated with electrical resistance produced the steam, introduced at the bottom of the glass extraction vessel (1000 mL). After steam passed through the plant material (positioned on a perforated plate set some centimeters away from the extraction vessel base), the steam and vaporized oil were condensed in a water-cooling system. Finally, TPEO and hydrosol (aqueous phase) were collected in a glass receiver (250 mL) equipped with a hydrosol overflow outlet and a water-cooling jacket to decrease the formation of artifacts due to overheating [33,34]. After separation by decantation, the TPEO was dried over anhydrous Na_2_SO_4_ and stored at −18 °C in sealed vials for future analysis (yielding 0.71% *v/w*). TPEO (%) yield was calculated and expressed in mL/100 g of dried plant material.

### 2.3. Gas Chromatography-Mass Spectrometry Analysis

Chromatographic analysis was carried out on a gas chromatograph (HP6890 Gas-Chromatograph) coupled with mass spectrometry (HP5973 Mass Spectrometer). One μL from the diluted TPEO (1/1000 *v/v*, in n-hexane) was injected in splitless mode. The sample was carried out at a helium flow rate of 1 mL/min through a Bruker BR-5MS fused silica capillary (Bruker, Billerica, MA, USA). The column was heated in a GC oven at a temperature range of 50 °C to 300 °C with 6 °C/min, the final hold was 5 min, and the solvent delay was 3 min. MS source was set at 230 °C and 150 °C for MS Quad, and ionization energy was 70 eV. The scanned compounds were weighted between 50 to 550 amu. All peaks from the obtained chromatograms represented chemical compounds found in the analyzed sample. The chemical constituents of TPEO were identified by comparison of their linear retention indices with those reported in this literature [35] and by computer matching NIST2.0 (USA National Institute of Science and Technology) library software.

### 2.4. Antimicrobial Activity

#### 2.4.1. Microbial Strains

The microbial strains used to determine the TPEO antibacterial and antifungal activity were reference strains: *Escherichia coli* ATCC 25922, *Shigella flexneri* ATCC 12022, *Streptococcus pyogenes* ATCC 19615, *Salmonella typhimurium* ATCC 14028, *Staphylococcus aureus* ATCC 25923, *Pseudomonas aeruginosa* ATCC 27853, *Candida albicans* ATCC 10231 and *Candida parapsilosis* ATCC 22019 (Microbiologics, Molsheim, France). According to the EFSA One Health 2020 Zoonoses Report [36], these strains generated the majority of foodborne outbreaks across the EU member states.

#### 2.4.2. Antibacterial Activity Assay

In the first stage, the antimicrobial effect of TPEO was determined by the Kirby–Bauer method based on the indications of the Clinical and Laboratory Standards Institute [37] and our previous studies [38]. From each standardized microbial suspension (0.5 MacFarland or approximately 10^8^ bacteria/mL and 2 × 10^6^ yeasts/mL), 100 µL were inoculated on Mueller–Hinton (MH) agar, or MHF (MH supplemented with defibrinated horse blood and β-nicotinamide adenine dinucleotide—bioMérieux, Marcy-l'Étoile, France). On the inoculated culture media, sterile blank disks (∅ 7 mm; BioMaxima, Lublin, Poland) were placed, impregnated with 10 µLTPEO; then, these plates were incubated for 24 h at 37 °C (for bacteria) and 28 °C (for *Candida* strains). The antimicrobial activity was reflected by the diameters of the inhibitory zones formed around the disks impregnated with TPEO. Gentamycin and fluconazole disks served as positive controls, while DMSO (dimethylsulphoxide) was used as the negative control. Experiments were performed in triplicate for each tested strain.

#### 2.4.3. Minimum Inhibitory Concentration (MIC)

Minimum inhibitory concentration (MIC) was determined by the broth microdilution method used according to the Clinical and Laboratory Standards Institute (CLSI) guidelines [39] for bacterial strains and the European Committee on Antimicrobial Susceptibility Testing (EUCAST) [40] for the *Candida* sp., respectively. The standardized inoculum was diluted, resulting in approximately 1–5 × 10^5^ colony-forming units/mL (CFU). Serial dilutions of TPEO in DMSO were obtained with concentrations of 40, 20, 10, 5, 2.5, 1.25, 0.625 mg/mL. Then, 100 µL of each TPEO dilution was added to 400 µL MH or MHF broth and 500 µL microbial suspension, resulting in a final microbial inoculum of 0.5 × 10^5^ CFU/mL. Following a 24 h incubation at 37 °C or 28 °C, the lowest concentration without visible growth was considered as MIC value. Tubes containing 100 µL DMSO, 500 µL microbial suspension, and 400 µL broth were used to control microbial growth. Experiments were performed in triplicate for each tested strain.

#### 2.4.4. Minimum Bactericidal Concentration (MBC) and Minimum Fungicidal Concentration (MFC)

The method described by Danciu et al. and Jianu et al. [38,41], with minor modifications, was employed to determine the MBC and MFC. Up to 1 µL from each test tube with no visible growth was inoculated on Columbia agar supplemented with 5% sheep blood and incubated for 24 h at 37 °C for bacterial strains or on Sabouraud Dextrose agar supplemented with chloramphenicol and incubated at 28 °C for *Candida* strains. The lowest TPEO concentration at which 99.5% of the inoculated microorganisms were killed was considered MBC and MFC, respectively [42,43]. Experiments were performed in triplicate for each tested strain.

### 2.5. Antioxidant Activity 

#### 2.5.1. Sample Preparation 

A total of 200 ppm (*v/v*) TPEO was added to 10 mL cold-pressed sunflower oil. Separately, 200 ppm (*w*/*v*) of two synthetic antioxidants (BHT and BHA), used as the positive controls, were added to 10 mL cold-pressed sunflower oil, according to Directive 2006/52/EC [44]. As a negative control, 10 mL of cold-pressed sunflower oil without any additive was utilized.

#### 2.5.2. Peroxide Value (PV)

The PV (meq of oxygen kg^−1^) was carried out by the potentiometric method every four days up to 24 days using the ISO 27107:2010 [45]. Experiments were performed in triplicate.

#### 2.5.3. Thiobarbituric Acid Value (TBA)

The TBA value (g malondialdehyde g^−1^) was carried out every four days up to 24 days, based on the previously described methods by Jianu et al. [46]. Briefly, each sample (2 g) was mixed with benzene (5 mL) and 0.67% TBA aqueous solution (4 mL). The mixtures were constantly shaken for 1 h using a mechanical shaker. Subsequently, the supernatant was incubated for 45 min in a boiling water bath. After cooling to ambient temperature, its absorbance was measured at 540 nm (Specord 210 Analytik Jena spectrophotometer). Experiments were performed in triplicate.

#### 2.5.4. Antioxidant Activity by 1,1-diphenyl-2-picrylhydrazyl (DPPH) Radical Scavenging Assay 

With minor modifications, the scavenging effect on DPPH radical was carried out by the method reported earlier by Brand-Williams et al. [47]. Briefly, serial dilutions of the TPEO (ranging from 1.5 mg/mL to 2.93 µg/mL) were prepared in pure methanol, then 100 μL of each of them were mixed with 10 μL from a DPPH stock solution of 1 mg/mL. The samples were added to the wells of a 96-well plate and stored at 25 °C for 30 min in the dark. The absorbance was registered at the wavelength of 515 nm (Tecan i-control, 1.10.4.0 infinite 200Pro spectrophotometer). The following formula computed the percentage of DPPH inhibition: % inhibition = (A_blank_ − A_sample_) 100/A_blank_ (A_blank_ and A_sample_ are the absorbances of the control and the absorbance of the test sample, respectively). Experiments were performed in triplicate with BHA, alpha-tocopherol and delta-tocopherol serving as positive controls and methanol as the negative control. BioDataFit 1.02 program (Chang Broscience Inc, Castro Valley, CA, USA) was employed to determine the IC_50_ (μg/L).

#### 2.5.5. Antioxidant Activity by [2,2′-azinobis(3-ethylbenzothiazoline-6-sulfonic Acid) diammonium] (ABTS) Radical Scavenging Assay 

ABTS radical cation was performed as reported by Rădulescu et al. [48]. Briefly, a fresh ABTS^+^ solution was prepared from 7 mM of ABTS at pH 7.4 (5 mM NaH_2_PO_4_, 5 mM Na_2_HPO_4_, and 154 mM NaCl) and 2.5 mM K_2_S_2_O_8_ (final concentration) and stored in the dark for 16 h at 25 °C. Before use, the ABTS^+^ solution absorbance was adjusted to 0.700 ± 0.021 at 734 nm [49]. Next, aliquots (100 μL) of the TPEO in methanol, with concentrations from 1.5 to 0.093 mg/mL, were added to the ABTS^+^ solution (1 mL) and vigorously mixed. After 10 min incubation (at 25 °C in the dark), the absorbance was measured at 734 nm. Experiments were performed in triplicate with BHA, alpha-tocopherol and delta-tocopherol serving as positive controls. BioDataFit 1.02 program (Chang Broscience Inc, Castro Valley, CA, USA) was employed to determine the IC50 (μg/L).

#### 2.5.6. Beta-Carotene/Linoleic Acid Bleaching Assay

The assay was performed as reported by Rădulescu et al. [48]. Briefly, a mixture of 0.5 mg *β*-carotene in 1 mL of chloroform, 200 mg Tween 40 and 25 μL linoleic acid was prepared. The chloroform was then evaporated under vacuum (at 40 °C for 5 min). Subsequently, the residue was diluted with 100 mL of 3% hydrogen peroxide aqueous solution to form a transparent yellowish emulsion. Finally, TPEO and BHA (serving as positive control) were individually dissolved in ethanol (2 mg ml^−1^), then 350 µL transferred in test tubes with 2.5 mL of the beta-carotene and linoleic acid mixture, stirred exhaustively, and incubated for 48 h at room temperature. The absorbance values were registered at 490 nm. Experiments were performed in triplicate. The Relative Antioxidant Activity (RAA %) was calculated as follows: RAA = A_TPEO_/A_BHA_, where A_TPEO_ and A_BHA_ are the absorption of TPEO and the absorbance of the BHA, respectively.

### 2.6. In Silico Prediction of Bioactivity and Molecular Docking Studies

Molecular docking simulations were carried out following in detail, the previously described method [42]. In brief, protein structures from the RCSB Protein Data Bank [50] (Table 1) were optimized as docking targets. Discovery Studio 4.1 was used to create 3D structures of the 39 TPEO components (Dassault Systems BIOVIA, San Diego, CA, USA). Molecular docking was carried out with the PyRx v0.8 virtual screening software (The Scripps Research Institute, La Jolla, CA, USA) and Vina’s encoded scoring function [51]. The target protein was docked with ten conformers of each input molecular structure. The calculated root means square deviation (RMSD) between the predicted and experimental native ligand docking poses for each case could not exceed a 2 Å threshold in order to validate our protocol. The docking grid box coordinates and size were chosen to best fit the active binding site (Table 1). Docking scores were generated as ∆G binding energy values (kcal/mol). Accelrys Discovery Studio 4.1 was also used to analyze protein-ligand binding interactions (Dassault Systems BIOVIA, San Diego, CA, USA).

### 2.7. Statistical Analysis

The antioxidant properties of TPEO were tested using ANOVA procedure followed by post-hoc analysis. Two-way ANOVA with main and interaction effects was performed in the case of MDA and peroxide values with sample and incubation period as main effects. Because the number of observations of sample per incubation period was low (only nine observations), the Shapiro–Wilk test for normality was applied on the ANOVA residuals. For the homogeneity of variances across groups, the Levene test was used. All the assumption checks regarding normality and homogeneity of variances, were successfully completed after the removal of two extreme outliers. Because the interaction effect was highly significant, the post-hoc analysis was performed at each incubation period in turn using Tukey’s parametric test. One-way ANOVA was applied on DPPH and ABTS with antioxidant type as grouping variable in both situations. The assumptions regarding the normality of data and homogeneity of variances were checked with the same procedure as described above. In post-hoc analysis, Tukey’s test was applied in the case of DPPH. In the case of ABTS, due to non-homogeneity of variances (Levene’s test, *p* < 0.001) the Games–Howell test was applied. A similar approach was used to test the antimicrobial properties of TPEO: one-way ANOVA applied on the diffusion disk diameter with bacteria as main effect followed by post-hoc analysis using the Tukey test. The significance level considered for all the above-mentioned testing procedures was 0.05. The statistical software used was Jamovi (Version 2.2.5).

## 3. Results. Discussion

### 3.1. TPEO Chemical Composition

The steam distillation of *T. pulegioides*’ dried aerial parts provided a brown yellow-colored oil with a 0.71% (*v/w*) yield and an agreeable odor reminiscent of *T. vulgaris* oil. Consistent results were obtained by Pavel et al. [14], that reported a yield of 0.7% (*v/w*) for EO obtained from *T. pulegioides* growing wild in Romania. Beicu et al. [52] reported that yields range from 0.44 to 0.49% (*v/w*) for Romanian *T. pulegioides.* Similar oil content has been reported for *T. pulegioides* growing wild in in Italy, 0.50 to 0.87% (*v/w*) [53] and Lithuania, 0.23 to 0.96% (*v/w*) [54]. Furthermore, the results compromise the European Pharmacopoeia requirements for *Serpylli herba* that mentioned a yield of at least 0.3% [55].

GC-MS analyses identified 39 components, representing 98.46% of the total identified compounds. Thymol (22.89%) is the main compound, followed by *para*-cymene (14.57%), thymol methyl ether (11.19%), isothymol methyl ether (10.45%), and beta-bisabolene (9.53%) (Table 2), suggesting that the sample belongs to the thymol chemotype. The thymol chemotype, rich in thymol and its precursors (*para*-cymene and gamma-terpinene), and derivatives (thymol methyl ether), was also reported in Germany, Poland, Hungary, and Italy [16,56,57,58]. In contrast with our findings, Pavel et al. [14] documented the carvacrol type for *T. pulegioides* growing wild in Romania. The amount and chemical composition of TPEO is influenced by the harvesting time [53], climatic factors [59], soil type [54], and the duration of hydrodistillation [60]. In addition, the plant parts employed significantly affect chemotypes and contribute to their relative abundance [53].

### 3.2. TPEO Antimicrobial Activity

The in vitro antimicrobial activity of the TPEO was qualitatively and quantitatively evaluated against eight strains of pathogenic bacteria and *Candida* spp. by the presence (or absence) of inhibition zones, diameters of inhibition zones, and MIC, MBC, and MFC values with the standard antimicrobial drugs gentamycin and fluconazole. DMSO was used as negative control, and the recorded antimicrobial effect of this compound was absent, therefore was not reported as separate set of results. The halo diameters of the inhibition zone caused by TPEO against the tested microorganism strains ranged between 15.3 ± 0.58 mm and 45.7 ± 2.52 mm (Table 3), suggesting that the oil exerts low to moderate antimicrobial effects. The results showed that *C. parapsilosis, C. albicans*, *S. pyogenes*, and *S. aureus* were more sensitive than the other microorganism tested. Our results agree with previous investigations [7,13,14,52], which recorded that TPEO displayed antimicrobial activity against *C. parapsilosis, C. albicans*, *S. pyogenes*, and *S. aureus*. The tested strains’ recorded MICs, MBCs, and MFCs were 1.25, 5, 10, and 20 mg/mL, respectively (Table 3). The TPEO exhibited a moderate MIC for *C. parapsilosis, C. albicans*, *S. pyogenes*, and *S. aureus* and showed low activity against the rest of the analyzed bacteria. Furthermore, our data is consistent with previous reports in which EOs exhibited comparable or higher antimicrobial activities, even when tested on drug-resistant bacterial strains such as methicillin-resistant *S. aureus* or carbapenem-resistant *P. aeruginosa* [61,62,63]. Overall, the Gram-positive strains appeared to be more susceptible than the Gram-negative. These differences might be generated by the presence of lipopolysaccharide molecules in the outer membrane of the Gram-negative bacteria cell wall that forms a hydrophilic permeability barrier providing protection against the effects of macromolecules and highly hydrophobic compounds [64,65,66]. The ability of these compounds to disrupt the permeability barrier of the bacterial cell walls, accompanied by the loss of chemiosmotic control, may represent the most likely explanation for the volatile oil’s lethal action [67].

### 3.3. TPEO Antioxidant Activity

**Figure 1:**
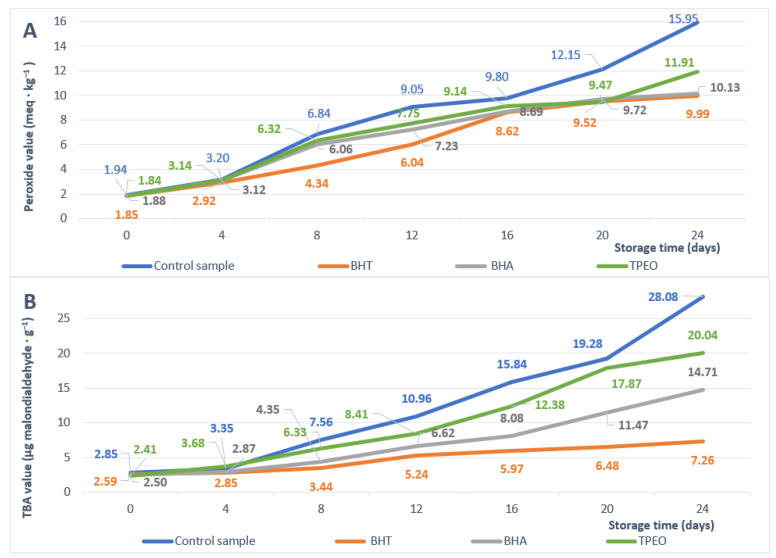
The influence of BHT, BHA, and TPEO on PVs (**A**) and TBA values (**B**) of sunflower oil samples during 24 days of storage. Values are expressed as means ± SD (n = 9).

Hydroperoxides are the primary products of lipid oxidation. Therefore, peroxide value can be employed as a direct marker for the primary phases of lipid oxidation. A higher peroxide value indicates lower oxidative stability of fats and oils during storage [68]. The antioxidant activity of TPEO has been carried out on cold-pressed sunflower oil. Figure 1A displays the progressive increase in PVs throughout the storage period of the studied sunflower oil samples. The PV for the sample treated with TPEO, at zero days incubation period, is not significantly different from samples treated with BHT (*p =* 0.979) and BHA (*p =* 0.402) according to the Tukey test. At four days, PV value for TPEO is not significantly different from the control sample (*p =* 0.407) and from BHA (*p =* 0.957). However, more promising results are found at 20 days of incubation, where PVs for the sample treated with TPEO are lower and significantly different than BHA values (*p <* 0.001) and lower but not significantly different than BHT values (*p =* 0.64).

The propagation phase generates considerable secondary reaction products, particularly malondialdehyde (MDA). Therefore, MDA concentration is a direct marker for the second phase of lipid peroxidation [69]. In addition, it has been suggested that MDA in food and edible oils has harmful effects on health [70]. Figure 1B displays the progressive increase in MDA throughout the storage period of the studied sunflower oil samples. The statistical analysis shows significant differences in MDA values between the sample treated with TPEO on one part and samples treated with BHA and BHT on the other part for all storage periods from 4 days through 24 days. Unfortunately, the MDA values for the sample treated with TPEO are higher than those treated with BHT and BHA. However, better results are recorded for the TPEO sample at zero days of incubation. More precisely, MDA values for the sample treated with TPEO are lower but not significantly different than MDA values for BHA (*p =* 0.252).

DPPH radical scavenging assay is a broadly used method for determining the antioxidant activity of natural products or synthetic compounds [71]. The procedure is based on reducing DPPH in solution with a hydrogen-donating antioxidant and is dependent on the formation of the nonradical form DPPH-H in the reaction [72]. TPEO reduced the stable free radical DPPH with an IC_50_ value of 19.13 ± 0.61 μg/mL (Table 4). The DPPH values for TPEO are significantly lower than for alpha-tocopherol (*p* < 0.001) and delta-tocopherol (*p <* 0.001), according to Tukey’s test. Previously, Fernandes et al. reported low antioxidant ability for *T. pulegioides* methanolic extract from a wild *T. pulegioides* (EC_50_ = 680 ± 30 μg/mL) [73], while Afonso et al. registered better results for *T. pulegioides* aqueous extracts (EC_50_ 9.50 ± 1.98 μg/mL) [74]. More recently, Kindl et al. reported promising results for a hydroethanolic extract of wild Croatian *T. pulegioides* (EC_50_ = 4.18 ± 0.02 μg/mL) [75]. However, no previous studies were recorded in the literature concerning the DPPH radical scavenging capacity of TPEO to permit us to make direct comparisons. Still, the DPPH radical scavenging ability reported herein for TPEO aligns with those previously described for *T. vulgaris*, *T. daenensis*, *T. serpyllum, T. linearis*, *T. sipyleus, T. longicaulis*, *T. algeriensis* and *T. polium* EOs [76,77,78,79,80,81].

The ABTS radical scavenging method is broadly used to analyze the antioxidant activity of single compounds and complex mixtures of various plants [82,83,84]. In the ABTS assay, TPEO indicated a strong antioxidant activity with an IC_50_ of 1.66 ± 0.1 μg/mL (Table 4), which was significantly (*p* < 0.001) more pronounced than that of alpha-tocopherol (IC_50_ 2.08 ± 0.1 μg/mL) and delta-tocopherol (*p* < 0.001) (IC_50_ 1.99 ± 0.1 μg/mL) according to the Games–Howell test. Our results are comparable with the findings of Taghouti et al. for *T. pulegioides* hydroethanolic extracts [85] and better than the findings for EO of *T. algeriensis, T. polium, T. vulgaris*, *T. broussonnettii*, *T. willdenowii, T. citriodorus* [80,86,87,88].

In the beta-carotene–linoleic acid bleaching assay, beta-carotene undergoes rapid discoloration without an antioxidant. Antioxidants can delay the extent of beta-carotene destruction by “neutralizing” the linoleate free radical or additional free radicals developed within the system [89]. Table 4 shows the inhibition of beta-carotene bleaching by the TPEO and the positive control (BHA). TPEO exhibited strong antioxidant activity (89.62 ± 0.14%) in the beta-carotene–linoleic acid test but lower than that of BHA (100%) (Table 4), with no significant differences (*p* > 0.05) observed. No previous studies were recorded in the literature regarding TPEO activity in the beta-carotene/linoleic acid system to allow us to make direct comparisons.

Given the preceding results, it is hard to ascertain the precise impact of each constituent on the antioxidant and antimicrobial activity of the essential oil. However, several of the TPEO’s major components, including thymol, thymol methyl ether, beta-bisabolene gamma-terpinene, p-cymene, or carvacrol, have previously been shown to exhibit potent antioxidant effects and antimicrobial properties on a variety of microbial strains [90,91,92,93,94], which can explain the overall synergistic effect of the TPEO observed in the present study.

### 3.4. In Silico Prediction of Bioactivity and Molecular Docking Studies

Computational methods are now an important tool for reducing the time required to unravel the action mechanisms of pharmacologically active substances. Molecular docking is a computational technique that allows the user to dock candidate molecules into the active site of a biological target and then classify the compound set based on their binding affinity, which is calculated using a scoring function [95]. Given that the essential oil demonstrated an antibacterial effect against two tested bacterial strains (*S. aureus, S. pyogenes*) and two tested fungal strains (*C. albicans*, *C. parapsilosis*), molecular docking was employed to establish potential target hits for the 39 components of TPEO. In addition, given its broad antioxidant versatility, using this strategy, we also intended to deduce potential targets that can be related to a protein-targeted antioxidant effect. For our current purpose, we used two sets of target proteins. The first set of proteins pertains to druggable targets that are frequently used for antimicrobial drug design and includes Isoleucyl-tRNA synthetase (IARS), DNA gyrase, d-alanine: d-alanine ligase (DDl), Streptococcus pneumoniae (DHPS), type IV topoisomerase, dihydrofolate reductase (DHFR), DNA gyrase subunit B, penicillin-binding protein 1a (PBP1a). The second set of targets consists of proteins that play critical roles in the metabolic generation of reactive oxygen species as byproducts and whose inhibition can reduce metabolic oxidative stress. This set includes lipoxygenase, CYP2C9, NADPH-oxidase, and xanthine oxidase. Table 5 displays the docking scores obtained for the 39 docked compounds.

**Figure 2:**
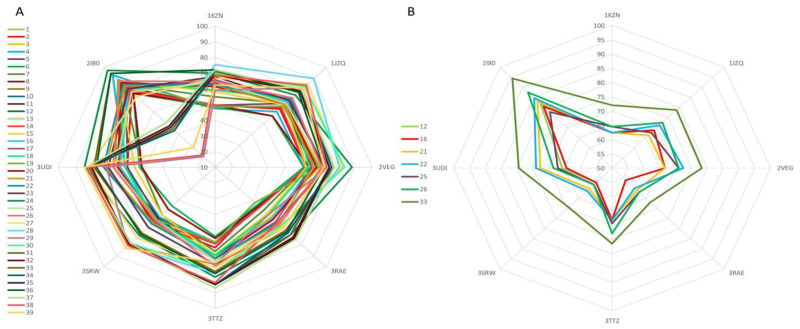
Graphical representation of the docking scores related to antimicrobial protein targets, corresponding to 39 TPEO components (**A**) and 7 major TPEO constituents (**B**) (representing over 75% of the oil); docking scores calculated as a percentage of the native ligand’s score of each target protein; the results are plotted in the form of a radar chart where docking scores (recalculated as a percentage of the native ligand’s docking score) of each compound, represent a series and the target proteins are in the corner of the graph.

The goal of our in silico-based method was to identify protein targets that can be inhibited by multiple components of our TPEO, or at least by the essential oil’s major constituents. Finding correlations within docking scores is difficult because each protein has different binding site characteristics and native ligands give different docking scores, resulting in each score set having different control values. To mitigate these inconveniences, we calculated each docking score as a percentage of its respective native ligand score (considered 100%). These percentages were represented as a radar graph, with the scores of each compound representing plot lines and the protein targets forming the corners of the radar chart. The final result should reveal the most likely targeted protein as a graph line stretch of the vast majority of all plotted series towards the protein targets corner. The graphical representation of docking scores for the antimicrobial protein targets are depicted in Figure 2A. When the scores of all 39 TPEO compounds are graphically represented, it is difficult to notice a tendency of a majority of the TPEO components to target a specific protein (Figure 2A). However, if we represent only the 7 major components (which account for more than 75% of the essential oil content) in the same graph, we can clearly see a tendency of the compounds to inhibit DDl (2I80) (Figure 2B). In this case, when compared to the native ligand (3-chloro-2,2-dimethyl-n-[4-(trifluoromethyl)phenyl]propenamides, −7.2 kcal/mol), the most active compounds were 24 (Linalyl anthranilate, −7 kcal/mol), 36 (beta-Sesquiphellandrene), and the major component 33 (beta-Bisabolene, -6.8 kcal/mol).

**Figure 3:**
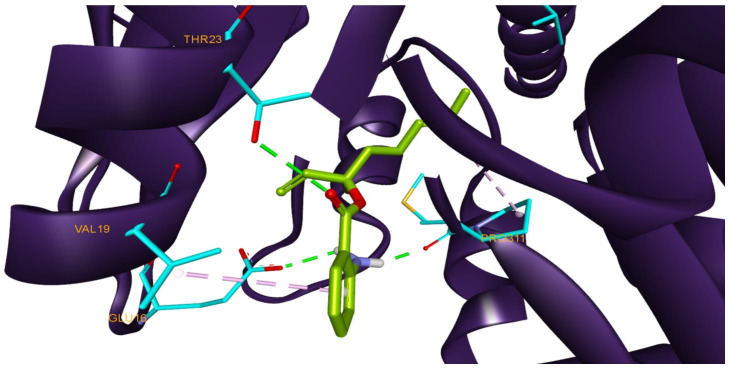
The structure of DDl (2I80) in complex with docked compound 24 (linalyl anthranilate) interacting with highlighted amino acids (cyan) Val19, Glu16, Pro311 and Thr23; HB interactions are depicted as green dotted lines and hydrophobic interactions as purple dotted lines.

Due to their crucial and universal involvement in bacterial cell-wall peptidoglycan production, which has been a proven target for antibiotics, DDl enzymes are a promising target for further chemotherapeutic research [96]. Based on known DDl structures, the conserved His, Val and Glu residues (His96, Val19, Glu16) have an essential role in the catalytic formation of d-alanyl-d-alanine. The native ligand of the DDl structure used for docking is an allosteric inhibitor that interacts with the aforementioned region within the binding site, rendering the proper catalytic activity of DDl ineffective. The only hydrogen bond (HB) formed by the native ligand is with Pro311 [97]. Similarly, compound 24 (linalyl anthranilate) interacts with the same binding site region and features the same HB with Pro311 making it a good candidate for DDl inhibition (Figure 3). Recent literature also revealed that cell membrane disruption and cell content leakage were two mechanisms related to linalyl anthranilate’s antibacterial effect against *K. pneumoniae* [98]. Terpene-rich essential oils exhibit antimicrobial activity and, due to their lipophilic structure, their bactericidal mechanism of action includes cell membrane disruption or impairment of cell membrane proteins [99]. Beta-bisabolene (compound 33) was also previously reported as an active compound against *S. aureus,* which can also restore the antibacterial effect of ampicillin against β-lactam resistant *S. aureus* strains [100]. While it is unclear that the mechanisms of action are related to bacterial cell membrane impairment, one of the TPEO mechanisms of action may correlate to the inhibition of DDl.

**Figure 4:**
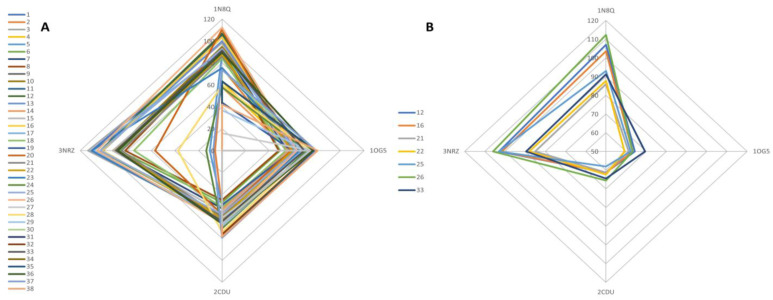
Graphical representation of the docking scores related to antioxidant protein targets, corresponding to 39 TPEO components (**A**) and 7 major TPEO constituents (**B**) (representing over 75% of the essential oil); docking scores calculated as a percentage of the native ligand’s score of each target protein; the results are plotted in the form of a radar chart where docking scores (recalculated as a percentage of the native ligand’s docking score) of each compound, represent a series and the target proteins are in the corner of the graph.

Using the same docking-based method, we also wanted to highlight a potential protein-targeted biological antioxidant effect of TPEO. By plotting all the 39 compounds’ docking scores, the graph shows that the majority of compounds tend to target two proteins: xanthine oxidase (3NRZ) and lipoxygenase (1N8Q) (Figure 4A). This targeted tendency towards the two proteins becomes much more significant when the chart only records docking scores of the 7 major compounds, previously mentioned (Figure 4B).

**Figure 5:**
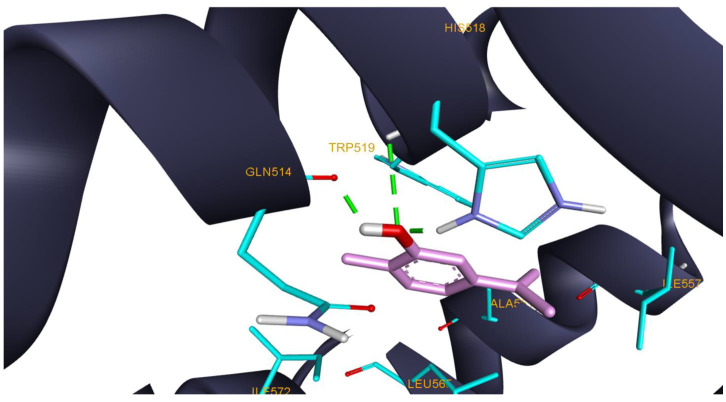
Structure of lipoxygenase (1N8Q) in complex with docked compound 26 (carvacrol) interacting with highlighted amino acids (cyan) Gln514, His518, and Trp519 through HB; hydrophobic interactions are omitted for better picture quality.

The oxidation reactions of polyunsaturated fatty acids catalyzed by lipoxygenase enzymes are one of several processes that generate reactive oxygen species (ROS) as byproducts. Lipoxygenases play an important role in pro-inflammatory signaling by regulating ROS levels [101]. Therefore, lipoxygenase inhibition can be therapeutically beneficial in both reducing ROS levels and the correlated inflammatory response. From the TPEO major components, compounds 12 (*para*-cymene), 16 (gamma-terpinene), and 26 (carvacrol) exhibited better docking scores when compared to the lipoxygenase native ligand. The most active in silico recorded compound was carvacrol. Ligand–protein interaction analysis shows carvacrol interacting with Glu802 and Ala1079 through HB and also forms other hydrophobic interactions with Phe914, Phe1009, and Ala1078 (Figure 5). These results are in line with previously reported studies that highlighted the anti-lipoxygenase activity of essential oils due to their carvacrol and *para*-cymene content or the anti-lipoxygenase activity of carvacrol alone [102,103,104,105].

**Figure 6:**
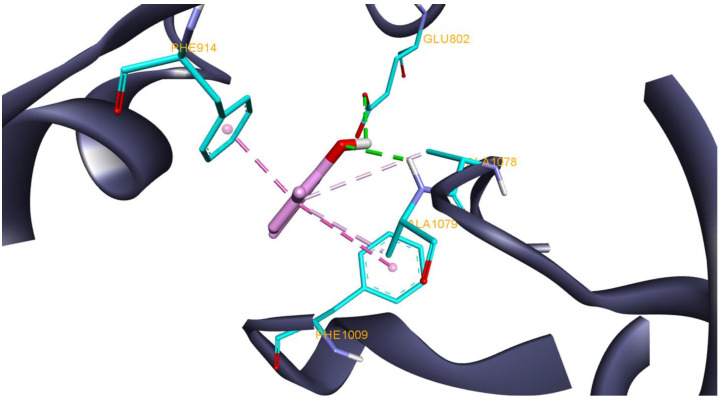
Structure of xanthine oxidase (3NRZ) in complex with docked compound 26 (carvacrol) interacting with highlighted amino acids (cyan) Glu802, Phe914, Phe1009, Ala1078 and Ala1079; HB interactions are depicted as green dotted lines and hydrophobic interactions as purple dotted lines.

Among the same major TPEO components, carvacrol also exhibited the highest in silico inhibitory activity against xanthine oxidase. Xanthine oxidase is a widely distributed enzyme that catalyzes the oxidation of hypoxanthine to xanthine and xanthine to uric acid. Mammalian xanthine oxidase is another physiological source of ROS, such as superoxide ion, hydrogen peroxide, and nitric oxide [106]. Ligand–protein interaction analysis revealed that carvacrol is tightly anchored within the protein binding pocket, mostly by hydrophobic interactions. Carvacrol also forms 3 HB with Gln514, His518, and Trp519 (Figure 6). Our results are in line with previously reported studies that highlighted both the in silico and in vitro inhibitory potential of carvacrol against xanthine oxidase [107,108]. Regarding those exposed, TPEO can be a significant source of chemicals with an antioxidant potential characterized by the suppression of ROS-producing proteins such as lipoxygenase and xanthine oxidase.

## 4. Conclusions

The research assessed the chemical composition, antibacterial and antioxidant activity of TPEO. The analyzed data shows that the volatile oil belongs to the thymol chemotype and exhibited good antimicrobial effects against *C. parapsilosis, C. albicans*, *S. pyogenes*, and *S. aureus*. The antioxidative data recorded reveal for the first time that TPEO, if compared to BHA in some specific conditions, inhibits primary and secondary oxidation products with the same efficacy, i.e., no statistically significant difference (*p* > 0.05), or even better with significant difference (*p* < 0.05). Moreover, TPEO antioxidant capabilities in DPPH and ABTS assays were better than those of alpha-tocopherol (*p* < 0.001) and delta-tocopherol (*p* < 0.001). Molecular docking-based in silico analysis revealed that one of the antibacterial mechanisms of TPEO could be attributed to DDl inhibition by both minor and major constituents. Docking determinations also revealed that major components of TPEO may potentially be biologically active antioxidant compounds by inhibiting ROS-producing enzymes such as lipoxygenase and xanthin oxidase. In conclusion, the results recommend TPEO as a new source of natural antioxidant and antibacterial agents with applicability in the food and pharmaceutic industries.

## Figures and Tables

**Table 1:** Docking parameters used for the in silico evaluation of the 39 TPEO components.

Protein	PDB ID	Grid Box Center Coordinates	Grid Box Size
*Thermus thermophilus* Isoleucyl-tRNA synthetase (IARS)	1JZQ	center_x = −27.8360428341	size_x = 17.8869815457
		center_y = 7.50432038116	size_y = 16.1154297919
		center_z = −29.4845039408	size_z = 11.3367245776
*Escherichia coli* DNA gyrase	1KZN	center_x = 19.6401666952	size_x = 17.2637959732
		center_y = 31.1030232133	size_y = 18.1500729831
		center_z = 34.3100514872	size_z = 16.6603783244
*Staphylococcus aureus* d-alanine: d-alanine ligase (DDl1)	2I80	center_x = 33.779502274	size_x = 10.5389819299
		center_y = 3.39522773577	size_y = 15.5864990289
		center_z = 25.2453856542	size_z = 9.85959076355
*Streptococcus pneumoniae* Dihydropteroate synthase (DHPS)	2VEG	center_x = 31.2676413349	size_x = 11.5588489508
		center_y = 48.9668812541	size_y = 18.1500729831
		center_z = −1.15410275954	size_z = 16.6603783244
*Streptococcus pneumoniae* type IV topoisomerase	3RAE	center_x = −33.7898737839	size_x = 15.0539568797
		center_y = 67.9013419953	size_y = 13.0375997543
		center_z = −23.60407688	size_z = 10.109792847
*Staphylococcus aureus* Dihydrofolate reductase (DHFR)	3SRW	center_x = −5.45640482801	size_x = 11.8275449768
		center_y = −31.6903217724	size_y = 13.023083456
		center_z = 6.17826267819	size_z = 9.9565989264
*Staphylococcus aureus* DNA gyrase subunit B	3TTZ	center_x = 17.0364358037	size_x = 20.0369846269
		center_y = −18.8857041496	size_y = 13.0029608168
		center_z = 6.75791376296	size_z = 10.7626967724
*Acinetobacter baumannii* Penicillin-binding protein 1a (PBP1a)	3UDI	center_x = 34.9732513158	size_x = 9.90891076337
		center_y = −0.0306900110435	size_y = 14.1836672574
		center_z = 12.3758077698	size_z = 11.2203132474
Lipoxygenase	1N8Q	center_x = 21.2593790904	size_x = 12.6872398333
		center_y = 1.76637901263	size_y = 11.4545838995
		center_z = 18.4081489959	size_z = 11.6795535545
CYP2C9	1OG5	center_x = −19.8764113706	size_x = 12.0869750978
		center_y = 87.4619136116	size_y = 11.4545838995
		center_z = 39.1269527463	size_z = 12.4186825691
NADPH-oxidase	2CDU	center_x = 19.720599136	size_x = 14.1566335714
		center_y = −6.31764559019	size_y = 13.6787417937
		center_z = −1.64253696973	size_z = 14.2233138143
Xanthine oxidase	3NRZ	center_x = 36.9278386062	size_x = 9.25016590812
		center_y = 20.1433878392	size_y = 9.63005270761
		center_z = 17.9487970315	size_z = 8.24844724947

**Table 2:** Chemical composition of the essential oil isolated from *T. pulegioides*.

No	Common Name	RI ^a^	Area %
1	Bicyclo [3.1.0]hexane, 4-methyl-1-(1-methylethyl), didehydro deriv	912	0.98
2	alpha-Pinene	918	0.57
3	2,4-Thujadiene	927	tr.
4	Camphene	933	0.31
5	beta-Pinene	959	0.41
6	3-Octanone	964	0.15
7	beta-Myrcene	970	0.88
8	3-Octanol	976	tr.
9	alpha-Phellandrene	987	0.22
10	4-Carene	990	0.08
11	alpha-Terpinene	998	1.60
12	*para*-Cymene	1006	14.57
13	d-Limonene	1011	0.26
14	Eucalyptol	1014	0.44
15	beta-cis-Ocimene	1029	0.07
16	gamma-Terpinene	1042	6.93
17	Terpinolene	1072	0.14
18	Linalool	1086	0.35
19	Borneol	1167	0.32
20	Terpinen-4-ol	1177	0.37
21	Thymol methyl ether	1233	11.19
22	Isothymol methyl ether	1244	10.44
23	Thymoquinone	1253	0.72
24	Linalyl anthranilate	1255	0.76
25	Thymol	1302	22.89
26	Carvacrol	1309	3.23
27	alpha-Copaene	1394	0.31
28	beta-Bourbonene	1402	0.47
29	beta-Caryophyllene	1440	4.80
30	beta-Cubebene	1450	0.29
31	gamma-Muurolene	1496	1.03
32	alpha-Muurolene	1520	0.26
33	beta-Bisabolene	1530	9.53
34	gamma-Cadinene	1534	0.76
35	delta-Cadinene	1540	1.66
36	beta-Sesquiphellandrene	1545	0.21
37	alpha-Calacorene	1561	0.26
38	Spathulenol	1595	0.27
39	Caryophyllene oxide	1600	0.73
		Total	98.46

^a^ The retention index (RI) was calculated using a homologous series of n-alkanes C_8_-C_20_.

**Table 3:** Antimicrobial activity of the TPEO by disk diffusion, MIC, MBC and MFC *.

Bacterial and Yeast Strains	Disk Diffusion (mm)	MIC Value (mg/mL)	MBC Value (mg/mL)	MFC Value (mg/mL)
*Streptococcus pyogenes* ATCC 19615	39.3(±0.58) ^ab^	1.25	1.25	n.t.
*Staphylococcus aureus* ATCC 25923	37.7(±1.53) ^b^	1.25	1.25	n.t.
*Escherichia coli* ATCC 25922	26.3(±1.16) ^cd^	5	10	n.t.
*Salmonella typhimurium* ATCC 14028	22.3(±0.58) ^c^	10	10	n.t.
*Shigella flexneri* ATCC 12022	27(±1.73) ^d^	10	10	n.t.
*Pseudomonas aeruginosa* ATCC 27853	15.3(±0.58) ^f^	20	20	n.t.
*Candida albicans* ATCC 10231	43(±2.65) ^a^	1.25	n.t.	1.25
*Candida parapsilosis* ATCC 22019	45.7(±2.52) ^e^	1.25	n.t.	1.25

* The diameter of the zone of inhibition is presented as means (n = 9) ± standard deviation, and the mean value for MIC, MBC and MFC; n.t. not tested; Values with different superscript are significantly different according to Tukey test, *p* < 0.05.

**Table 4:** Yield, total phenolic content and DPPH radical scavenging activities of the essential oil extracted from *T. pulegioides*.

Parameter	TPEO	BHA ^1^	alpha-Tocopherol	delta-Tocopherol
Yield (%)	0.71	-	-	-
DPPH, IC_50_ (μg/mL)	19.13(±0.61) ^d^	8.11(±0.45) ^b^	23.93(±0.43) ^a^	22.77(±0.49) ^c^
ABTS, IC_50_ (μg/mL)	1.66(±0.1) ^c^	0.71(±0.03) ^b^	2.08(±0.1) ^a^	1.99(±0.1) ^a^
beta-carotene/linoleic acid, RAA ^2^ (%)	89.62(±0.14)	100	n.t.^3^	n.t.^3^

^1^ BHA—butylated hydroxyanisole; ^2^ RAA—relative antioxidative activity; ^3^ n.t.—not tested; values with different superscript are significantly different according to Tukey/ Games–Howell test, *p <* 0.05.

**Table 5:** Docking scores for compounds 1–39 (binding energy, ∆G kcal/mol); compounds with better docking scores as compared to the target’s native ligand score are highlighted in yellow.

Target PDB ID	1JZQ	1KZN	2I80	3RAE	3SRW	3TTZ	3UDI	1N8Q	1OG5	2CDU	3NRZ
Docked Compound ID	Binding Free Energy ∆G (kcal/mol)										
Native ligand	−8	−9.3	−7.2	−10	−9.8	−7.8	−7.3	−5.7	−9.8	−9.3	−6.7
1	−5.5	−5.9	−6.1	−5.6	−5.7	−5.7	−4.8	−5.6	−6.2	−6	−7.4
2	−5	−4.6	−5.6	−4.4	−5.9	−5.2	−4.4	−4.3	−5.5	−4.8	−0.4
3	−5.4	−6	−6	−5.5	−5.6	−5.6	−4.8	−5.6	−5.8	−5.9	−7.6
4	−4.8	−4.4	−6.7	−4.6	−5.4	−4.9	−4.5	−3.6	−5.7	−4.7	0.4
5	−5.4	−4.6	−5.9	−4.4	−5.5	−5	−4.4	−5	−5.5	−4.7	−0.7
6	−4.5	−4.5	−5.5	−4.4	−4.4	−4.6	−3.9	−5.3	−4.8	−4.4	−5.8
7	−5.4	−5.1	−6.1	−4.9	−5.2	−5.4	−3.8	−5.4	−5.4	−4.9	−6
8	−4.5	−4.6	−5.5	−4.5	−4.7	−4.7	−3.8	−5.2	−4.7	−4.2	−5.5
9	−5.5	−5.8	−5.7	−5.6	−5.6	−5.8	−4.7	−5.9	−6.2	−5.7	−6.8
10	−5.3	−5.9	−6.1	−5.7	−5.9	−5.6	−4.7	−5.4	−6.2	−5.6	−6.1
11	−5.5	−5.8	−5.9	−5.6	−5.5	−5.8	−4.8	−6	−6.3	−5.7	−6.8
12	−5.5	−5.8	−5.9	−5.6	−5.6	−5.8	−4.8	−6.1	−6.3	−5.7	−6.9
13	−5.4	−5.8	−5.7	−5.6	−5.6	−5.8	−4.7	−5.7	−6.3	−5.7	−6.8
14	−5.1	−4.6	−5.7	−4.6	−5.8	−5	−4.8	−3.3	−5.5	−5	3.4
15	−5.3	−5.4	−6.3	−5.2	−5.2	−5.6	−4.1	−5	−5.6	−5.2	−6.2
16	−5.5	−5.8	−5.8	−5.6	−5.6	−5.8	−4.7	−5.9	−6.2	−5.7	−6.8
17	−5.6	−6.1	−5.9	−5.9	−5.8	−5.9	−4.9	−5.2	−6.6	−5.9	−7.3
18	−5.7	−5.5	−6.3	−5.3	−5.7	−5.6	−4.5	−4.8	−5.3	−4.8	−5
19	−5.2	−4.5	−6.4	−4.2	−5.6	−4.9	−4.9	−2.5	−5.8	−4.6	2.7
20	−5.4	−5.7	−6.2	−5.7	−5.9	−5.8	−4.8	−6.3	−5.7	−5.4	−3.8
21	−5.3	−5.8	−6	−6.1	−5.9	−5.8	−5.3	−4.9	−6	−5.7	−5.7
22	−5.7	−5.8	−6.1	−6	−6	−5.8	−5.4	−5	−5.8	−5.8	−5.9
23	−5.6	−6.3	−5.8	−6.5	−6.3	−6.1	−5.2	−4.3	−6.4	−6	−7.4
24	−6	−6.5	−7	−7.1	−7	−6.8	−6.2	−3.3	−7	−6.6	−0.9
25	−5.4	−6	−5.6	−6.2	−5.7	−5.9	−4.9	−5.3	−6.1	−5.4	−6.9
26	−5.8	−6	−6.3	−6.2	−5.7	−6.2	−5	−6.4	−6.2	−6.1	−7.1
27	−6.8	−6.2	−5.3	−6.4	−7.2	−6.3	−6	−0.9	−7.4	−5.7	1.2
28	−7.2	−7	−3.3	−6.9	−7.9	−6.6	−5.9	−3.4	−7.2	−6.9	−2.5
29	−6.5	−5.5	−1.4	−5.3	−7.9	−6.1	−5.5	−2.1	−7.2	−6.4	2.5
30	−6.6	−6.5	−3.1	−6.3	−7.7	−6.5	−6	−4.2	−7.4	−6	4.6
31	−6.4	−6.6	−3.2	−7.1	−7.8	−7.2	−6	−3.6	−7.6	−7.2	0.5
32	−6.3	−6.4	−3.3	−7.3	−7.8	−7.2	−5.9	−3.6	−7.6	−7.1	1.2
33	−6.3	−6.7	−6.8	−6.7	−6.8	−6.5	−5.8	−5.2	−6.8	−6	−6
34	−6.4	−6.7	−3.1	−6.9	−7.7	−7.2	−6.1	−3.6	−7.6	−7.3	0.4
35	−6.3	−6.7	−3.4	−7.4	−7.7	−7.2	−6.1	−3.6	−7.7	−7.3	0.2
36	−6.2	−6.7	−6.8	−6.8	−6.9	−6.6	−5.7	−5.1	−7.5	−6.2	−5.9
37	−6.4	−6.7	−3.6	−7.5	−7.7	−7.4	−6.1	−4.2	−7.8	−7.4	−0.5
38	−6.7	−6.4	−1.5	−6	−7.9	−7.1	−6.1	−2.4	−7.9	−7.3	4
39	−6.5	−5.8	−2	−6.5	−8.1	−6	−6.2	1.2	−7.3	−6.2	3.7

## Data Availability

Data are contained within the article.
